# Innovative decision making tools using discrete mathematics for stress urinary incontinence treatment

**DOI:** 10.1038/s41598-024-60407-w

**Published:** 2024-04-30

**Authors:** Nobuo Okui

**Affiliations:** 1 Kanagawa Dental University, 82 Inaka Cho, Yokosuka, Kanagawa 238-8580 Japan; 2 Yokosuka Urogynecology and Urology Clinic, 2-6 Ootaki, Yokosuka, Kanagawa 238-0008 Japan

**Keywords:** Urology, Bladder, Urethra, Urinary incontinence, Urological manifestations, Outcomes research

## Abstract

In this study, we applied graph theory to clinical decision-making for Stress Urinary Incontinence (SUI) treatment. Utilizing discrete mathematics, we developed a system to visually understand the shortest path to the desired treatment outcomes by considering various patient variables. Focusing on women aged 35–50, we examined the effectiveness of Tension-free Vaginal Tape (TVT) surgery and Vaginal Erbium Laser (VEL) treatment for over 15 years. The TVT group consisted of 102 patients who underwent surgery using either the Advantage Fit mid-urethral sling system (Boston Scientific Co., MA, USA) or the GYNECARE TVT retropubic system (Ethicon Inc., NJ, USA). The VEL group included 113 patients treated with a non-ablative Erbium: YAG laser (FotonaSmooth™ XS; Fotona d.o.o., Ljubljana, Slovenia), and there were 112 patients in the control group. We constructed a network diagram analyzing the correlations between health, demographic factors, treatment methods, and patient outcomes. By calculating the shortest path using heuristic functions, we identified significant correlations and treatment effects. This approach supports patient decision making by choosing between TVT and VEL treatments based on individual objectives. Our findings provide new insights into SUI treatment, highlighting the value of a data-driven personalized approach for clinical decision-making. This interdisciplinary study bridges the gap between mathematics and medicine, demonstrating the importance of a data-centric approach in clinical decisions.

## Introduction

Graph theory, a central concept in discrete mathematics, focuses on noncontinuous data and structures. This powerful tool is used to represent and analyze the relationships between nodes^1,2^. Graph theory is used in a wide range of fields, from modern navigation systems such as Google Maps to air traffic control and robotic operations, contributing to efficient decision making by computing optimal routes and solutions^3,4^. However, the application of graph theory in clinical medicine, particularly in treatment selection, is largely unexplored, with our work being one of the few in this field^5^. This scarcity of research highlights the significant potential and need for further investigation in this area^5,6^.

This study aimed to develop a graph theory-based navigation system using data from previous research, focusing on the treatment choices for Stress Urinary Incontinence (SUI). SUI affects a significant proportion of women, significantly diminishing their quality of life^7–10^. A common treatment is mid-urethral sling (MUS) procedures using polypropylene mesh, but this method can lead to postoperative pain, foreign body reactions, inflammation, and even the emergence or worsening of Urgency Urinary Incontinence (UUI)^11–13^. Given these issues, Vaginal Erbium Laser (VEL) therapy has gained attention as a non-ablation alternative^14–18^. However, owing to the lack of sufficient data, choosing between MUS and VEL remains challenging. Our previous study compared Tension-free Vaginal Tape (TVT), a form of MUS, with VEL, but explaining these options to patients requires an understanding of complex statistical data^19^. The successful development of a navigation system that provides intuitive visual information would enable patients to make better-informed decisions regarding their treatment options, potentially enhancing quality of SUI treatment.

## Methods

### Ethical approval

This study is a post-analysis of our previous research and has been approved by the ethics committee of the Yokosuka Urogynecology and Urology Clinic^19^. All methods were performed in accordance with relevant guidelines and regulations, as confirmed by the principal author. All the participants provided written informed consent.

### Database

The study population consisted of women aged 35–50 years who met any of the following criteria: (1) underwent TVT surgery, (2) received VEL treatment, or (3) did not receive SUI treatment at multiple medical institutions (control group). The study was conducted over 15 years, from 2004 to 2019, and the subjects had a minimum of one year of follow-up data post-treatment. The treatment method (VEL or TVT) was chosen by patients after consultation, during which both treatment options were explained in detail. The sample size for each of the three groups was estimated to ensure a 95% confidence interval with a 5% margin of error using a tool from Raosoft, Inc. (Washington, USA).

Patients were selected based on the following criteria: (1) availability of 1-h pad test results before and one year after treatment^19^. (2) Completion of the International Consultation on Incontinence Questionnaire—Short Form (ICIQ)^19^. (3) Completion of the Overactive Bladder Symptom Score (OABSS)^20^. (4) Availability of detailed medical records before treatment (including information on age, Body Mass Index: BMI, marital status, childbirth history, desire for children, hypertension, diabetes, stroke, hyperlipidemia, etc.)^18–24^. (5) Presence of stroke, smoking history, spinal disease, pelvic surgery history, and menopausal disorders^18–24^.

The exclusion criteria were as follows: (1) patients hospitalized for a year for diseases other than SUI^18–24^. (2) Patients continuously undergoing hormone replacement therapy^18–24^. (3) Presence of bladder, uterine, or rectal prolapse^18–24^. (4) Use of overactive bladder (OAB) treatment drugs or anticholinergics^18–24^.

### Treatment methods and analysis process

In this study, routine pelvic floor muscle training (PFMT)^19^ was conducted under the supervision of urology specialists for all patients across the three groups. For the control group, the study duration was set as 1 year from the date of the first lesson, which was considered the starting day (day 0).

TVT surgery^10^ was performed under lumbar or general anesthesia using either the Advantage Fit™ mid-urethral sling system (Boston Scientific Co., MA, USA) or the GYNECARE TVT™ retropubic system (Ethicon Inc., NJ, USA). During this procedure, a 1 cm wide polypropylene mesh tape was inserted by a specialist and accurately positioned using transvaginal ultrasonography. The study was conducted over a year, starting on the day of surgery (day 0).

VEL^10^ was performed based on a common protocol across all facilities using a non-ablative Erbium: YAG laser (FotonaSmooth™ XS; Fotona d.o.o., Ljubljana, Slovenia). The treatment involved spraying 9% xylocaine on the external genitalia and vagina, followed by the insertion of a specialized glass tube into the vagina. A special long-pulse mode at a wavelength of 2940 nm was used for irradiation for 20 min. The entire anterior vaginal wall was irradiated for 10 min at 6 J/cm^2^, entire vagina for 5 min at 3.0 J/cm^2^, and periurethral area for 5 min at 10 J/cm^2^. This laser treatment was conducted over three sessions at two-month intervals. The study period for VEL was one year from the date of the first treatment, with this date as the starting day (day 0).

Throughout the study period, the settings and treatment processes for both sling surgery and VEL were consistent across all facilities.

The primary evaluation metric was a 1-h pad test result of 1 g or less at one year post-treatment, which was considered indicative of urinary incontinence cure^3^. Secondary evaluation metrics included improvements in total ICIQ and OABSS scores.

### Construction of a network diagram based on variable correlations

In this study, a dataset that included individual variables from previous research was used. This dataset comprised indicators such as TVT (a), VEL (b), PFMT (c), smoking status (d), occurrence of breast cancer (e), age (f), marital status (g), number of childbirths (h), hypertension (i), diabetes (j), obesity (k), stroke (l), hyperlipidemia (m), menopausal status (n), pelvic surgery (o), infertility treatment (p), spinal nerve issues (q), and desire to have children (r). Changes (Δ) in the 1-h pad test scores (s), ICIQ scores (t), and OABSS (u) were calculated from pre-treatment to one year post-treatment and included as variables.

In constructing the network diagram, direct correlations between TVT (a), VEL (b), and PFMT (c) were excluded from the analysis. This exclusion aimed to avoid redundancy and focus on the broader relationships between a wider range of variables.

To identify the strength of relationships between variables, a comprehensive correlation analysis was conducted using Python's statistical libraries. The correlation coefficients for each pair of variables were calculated and used to determine the proximity of variables within the network diagram.

Pearson's correlation coefficients and Spearman's rank correlation coefficients were calculated to examine the relationships between variables. The Pearson coefficient indicates the strength of a linear relationship when variables are normally distributed. Its values range from + 1 (a perfect positive relationship) to − 1 (a perfect negative relationship), with 0 indicating no relationship. The Spearman coefficient, based on rank data, indicates the strength of a relationship without assuming a normal distribution^25^.

The network diagram was generated using Python’s ‘networkx’ and ‘matplotlib’ libraries. These libraries provide force-directed graph-drawing algorithms for visualizing mechanical relationships between nodes. The variables were represented as nodes, and the edges between them were weighted according to the calculated correlation coefficients. The shorter the edge, the higher the correlation coefficient, which visually represents the strength of the relationship between variables^5^.

### Methodology for calculating the shortest path

In this study, the problem of calculating the shortest path from "s: Δ1-hour pad test" to "u: ΔOABSS" was analyzed to evaluate specific treatment effects. It was mandatory for all explored paths to include the intervention "c: PFMT". Additionally, the goal was to determine whether the treatment option "a: TVT" or "b: VEL" was more efficient.

A search algorithm utilizing heuristic functions has been employed to solve this problem^26^. The heuristic function calculates the estimated cost from the current node to the target node, thereby guiding the search algorithm to determine the shortest path more quickly^26^.

Specifically, using Python’s ‘networkx’ library, a weighted undirected graph was constructed comprising nodes (variables) and edges (relationships based on correlations between variables). Then, applying the A* algorithm with a custom heuristic function, the search included the mandatory node "c: PFMT" while conditionally incorporating treatment options "a: TVT" or "b: VEL".

This algorithm prioritizes the most promising paths based on a heuristic function, ultimately finding the shortest path that meets the necessary conditions^26^. In scenarios without additional factors, the heuristic function utilizes a simple linear estimate, thereby reducing the computational costs in the calculation of the shortest path.

The code for the heuristic function is as follows:
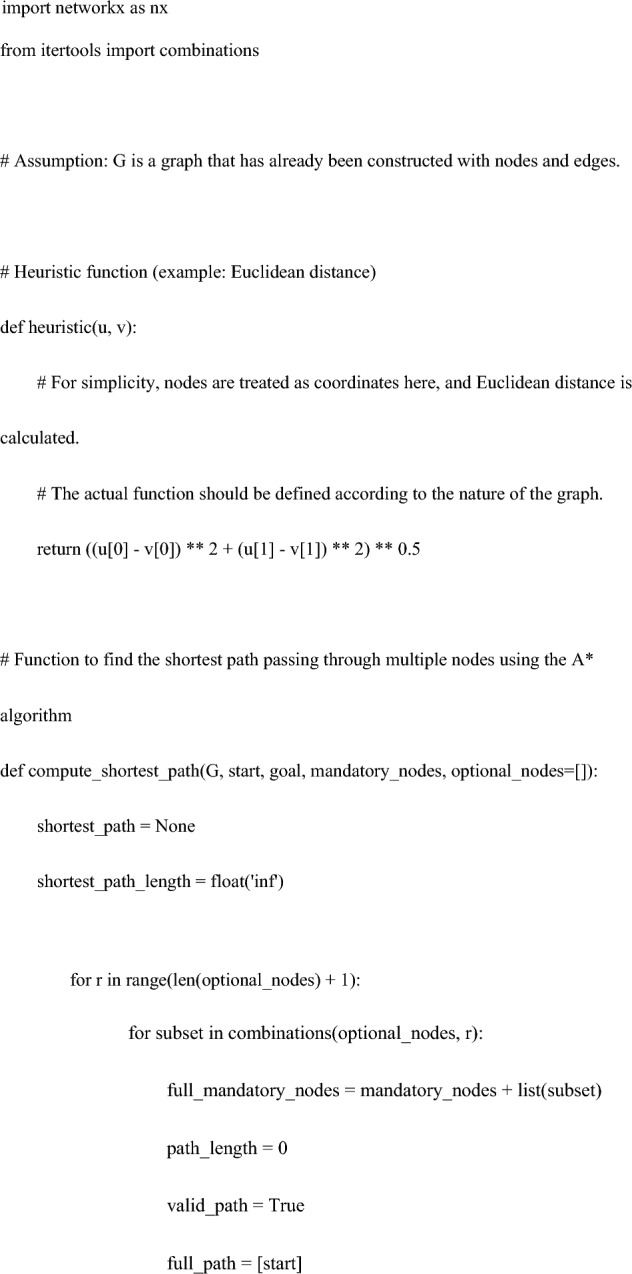

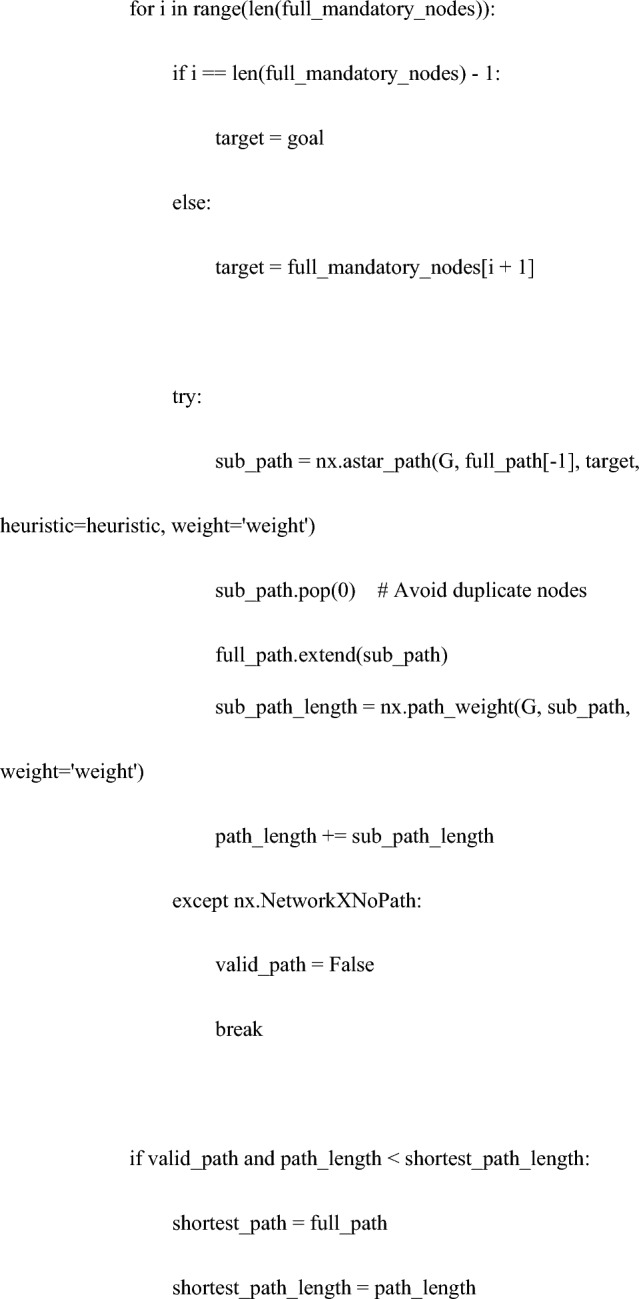

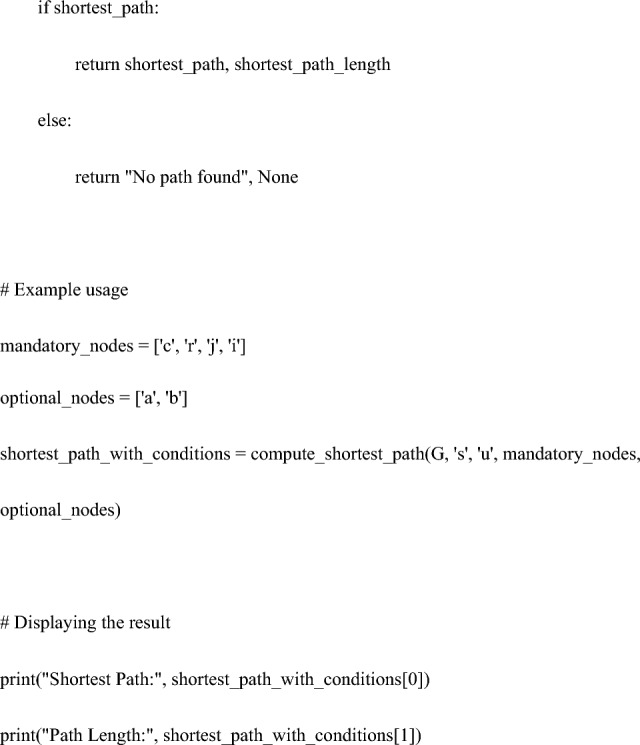


### Statistical analysis

In this study, the Kruskal–Wallis test was used for comparisons between the three groups (TVT, VEL, and control groups). For comparisons between the two groups (TVT and VEL), the Mann–Whitney U test was employed. Statistical software was programmed in ChatGPT (OpenAI, San Francisco, CA, USA) and implemented in Python (Python Software Foundation, Beaverton, OR, USA).

## Results

### Patient characteristics

In previous research, patient characteristics were analyzed in detail for 102 individuals in the TVT group, 113 in the VEL group, and 112 in the control group. The average age was 42.5 years (range 35–48 years) in the TVT group, 42.7 years (range 37–49 years) in the VEL group, and 43.3 years (range 38–48 years) in the control group. The average BMI was 23.2 kg/m^2^ in the TVT group, 22.9 kg/m^2^ in the VEL group, and 22.8 kg/m^2^ in the control group. No significant differences were observed in the proportion of married patients or partners across groups. However, significant differences were noted between the TVT and VEL groups regarding the desire for children and number of childbirths. 11.8% of patients in the TVT group expressed a desire for children, compared to 50.4% in the VEL group, a difference that was statistically significant (p < 0.001). Regarding the number of childbirths, the average for the TVT group was 1.3, whereas it was 1.1 for the VEL group, a difference that was also statistically significant (p = 0.015).

This prior study suggested that there were notable differences between the TVT and VEL groups, especially in terms of the desire for children and number of childbirths, potentially influencing treatment choices.

Table 1 shows a comparison of baseline characteristics among the three groups, highlighting that age, BMI, marital status, and baseline OABSS were comparable. Conversely, the TVT and VEL groups exhibited significant differences in their desire for children and number of childbirths.

**Table 1:** Demographics and populations of the three treatment groups.

Parameter	TVT group (n = 102)	VEL group (n = 113)	Control group (n = 112)	p-value*	p-value**
				(Total)	(TVT vs. VEL)
Age (years)	42.5 (35–48)	42.7 (37–49)	43.3 (38–48)	0.275	0.417
Married (Partner)	76.5%	73.5%	75.0%	0.878	0.612
No. of deliveries	1.3 (0–4)	1.1 (0–4)	1.3 (0–4)	0.015	0.015
BMI (kg/m^2^)	23.2 (19–25.5)	22.9 (20–25.6)	22.8 (20–25.6)	0.366	0.178
Menopause	11.8%	11.5%	8.9%	0.754	0.954
Desire for children	11.8%	50.4%	42.9%	< 0.001	< 0.001
Diabetes	2.0%	1.8%	3.6%	0.635	0.922
Hypertension	0.9%	1.8%	1.8%	0.863	0.627
Hyperlipidemia	2.0%	1.8%	3.6%	0.863	0.627
Cerebral infarction	1.0%	1.8%	1.8%	0.863	0.627
Smoking	13.7%	13.3%	13.4%	0.995	0.925
Spinal disease	0%	0%	0%	1.0	1.0
Breast cancer	0%	0%	0%	1.0	1.0
Pelvic surgery	2.0% 2 ovarian cysts	2.7% 1 ovarian cyst 1 uterine cancer 1 uterine fibroid	1.9% 1 ovarian cyst 1 uterine cancer	0.894	0.74
1-h pad test	31.6 g (15–60 g)	29.9 g (14–60 g)	34.3 g (12–62 g)	0.128	0.054
ICIQ	12.1 (8–21)	11.2 (7–21)	12.0 (8–21)	0.0924	0.0612
OABSS	1.83 (0–10)	2.24 (0–11)	1.7 (0–10)	0.892	0.821

Mean, minimum, and maximum values are shown for age, body mass index, number of deliveries, 1-h pad test, ICIQ, and OABSS. Percentages of patients taking medication for hypertension, diabetes, cerebral infarction, and hyperlipidemia are shown. Percentages of patients with a history of pelvic surgery and the names of the main diseases (number of patients) are shown.

*The Kruskal–Wallis test was used to compare the three groups (TVT, VEL, and control groups).

**The Mann–Whitney U test was used to compare the two groups (TVT and VEL groups).

### Network diagram

**Figure 1:**
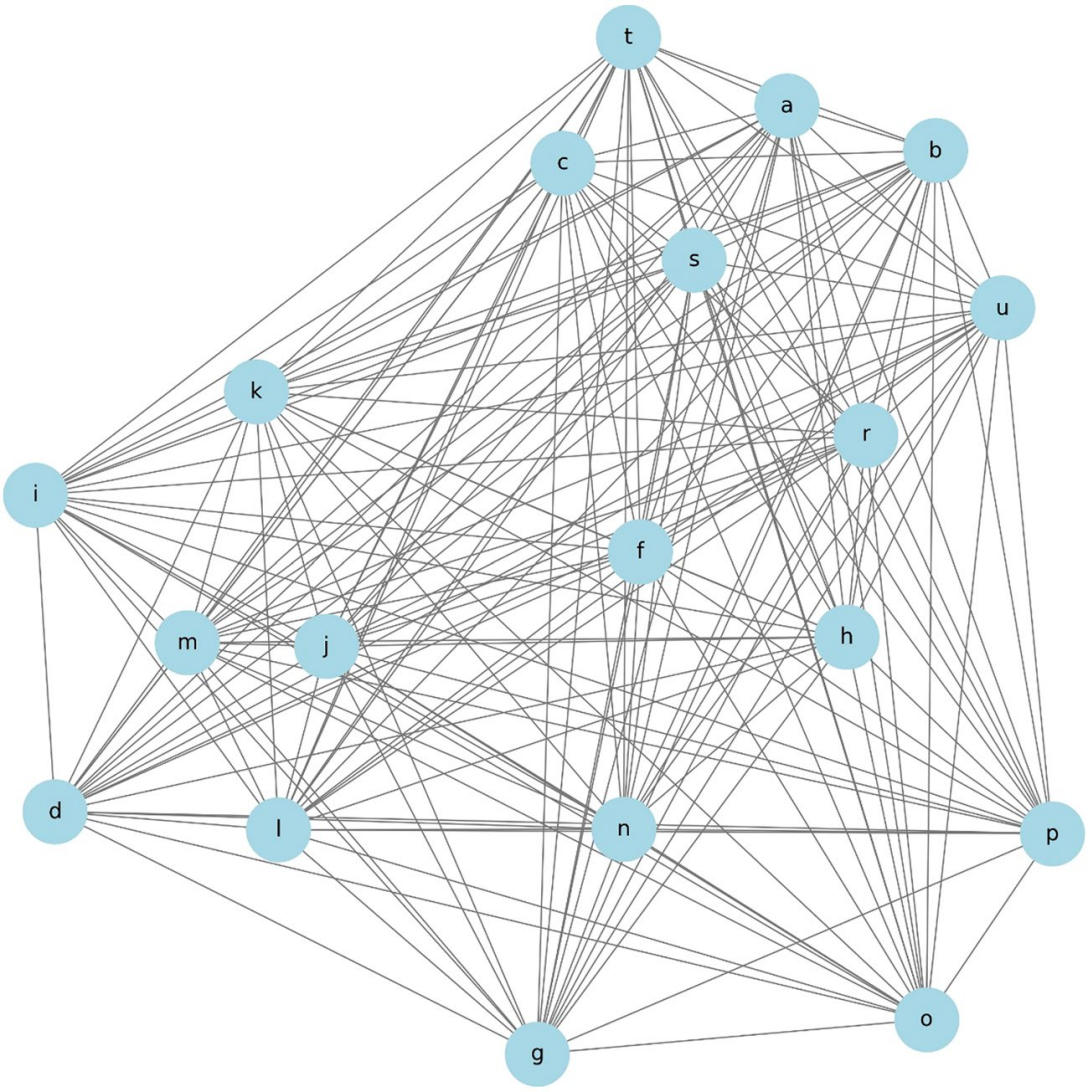
Network diagram. Each variable is represented as follows: a: TVT, b: VEL, c: PFMT, d: smoking, e: Breast Cancer, f: age, g: married, h: childbirth, i: hypertension, j: diabetes, k: obesity, l: stroke, m: hyperlipidemia, n: menopause, o: Pelvic Surgery, p: Infertility Treatment, q: Spinal Nerve, r: Desire for Children, s: Δ1-hour pad test, t: ΔICIQ, and u: ΔOABSS. The edges connecting the variables were shorter when the correlation coefficients were higher, indicating a stronger relationship. The total correlation coefficients for combinations a–b, a–c, and b–c were not calculated.

This figure presents a network diagram illustrating the correlations between various health and demographic variables in medical studies. The network analysis based on variable correlations revealed that specific variables are represented by central nodes, with strong correlations becoming apparent. Figure 1 depicts the network diagram showing the interactions among these variables. The variables are represented as nodes, and the length of the edges is determined based on the inverse of the correlation coefficients. Table 2 shows a summary of the correlation coefficients.

**Table 2:** Correlation between variable A and variable B.

Variable A	Variable B	Correlation
Diabetes	Hyperlipidemia	1.000
T121-hour pad test	T12ICIQ	0.915
Δ1-hour pad test	ΔICIQ	0.852
T0OABSS	T12OABSS	0.803
Cerebral Infarction	Hyperlipidemia	0.787
Diabetes	Cerebral Infarction	0.787
T0ICIQ	T12OABSS	0.748
T01-hour pad test	T0OABSS	0.748
T0ICIQ	T0OABSS	0.703
T01-hour pad test	T12OABSS	0.673
ΔICIQ	T12ICIQ	− 0.815
ΔICIQ	T121-hour pad test	− 0.763
Δ1-hour pad test	T121-hour pad test	− 0.724
Δ1-hour pad test	T12ICIQ	− 0.708
Age	Desire to Have a Baby	− 0.375
ΔOABSS	T12ICIQ	− 0.192
ΔOABSS	T121-hour pad test	− 0.177
Pelvic surgery	T01-hour pad test	− 0.154
Childbirth	ΔOABSS	− 0.146
Desire to have a baby	ΔOABSS	− 0.133

Age (f), obesity (k), and diabetes (j) showed strong correlations with many other variables. Notably, the correlation coefficient between diabetes (j) and hyperlipidemia (m) was 1.000, indicating a perfect positive correlation and suggesting these conditions often coexist. Stroke (l) also strongly correlated with both hyperlipidemia (m) and diabetes (j), with a correlation coefficient of 0.787.

The desire to have children (r) was significantly related to the outcomes of urinary incontinence tests (s: Δ1-hour pad test, t: ΔICIQ, u: ΔOABSS). These relationships are represented with short edges on the network diagram due to high correlation coefficients.

Marital status (g) showed significant correlations with hypertension (i), obesity (k), and hyperlipidemia (m), suggesting that health conditions related to lifestyle are mutually influenced by social situations.

The analysis revealed strong correlations between the outcomes of urinary incontinence tests before and after a certain period (denoted as T12 1-h pad test and T12 ICIQ), with a correlation coefficient of 0.915. This suggests a highly consistent change in these parameters over the observed period. Additionally, changes in the 1-h pad test and ICIQ scores (Δ1-hour pad test and ΔICIQ) were strongly correlated with a coefficient of 0.852, indicating that these measures are likely to vary together in response to interventions or over time.

A significant positive correlation was also noted between the initial and follow-up OABSS scores (T0 OABSS and T12 OABSS), with a coefficient of 0.803. This underscores the consistency in the pattern of symptoms related to overactive bladder syndrome as captured by these scores.

This network analysis clearly quantified the relationships between variables, visually demonstrating how each variable is interrelated. Strong correlations represent important factors in clinical decision-making and patient management. On the other hand, weak correlations suggest that these variables are also influenced by other factors.

### Thought Experiment 1

The examination focused on determining whether treatment a: TVT or b: VEL is recommended when aiming to improve the s: Δ1-hour pad test outcomes.

**Figure 2:**
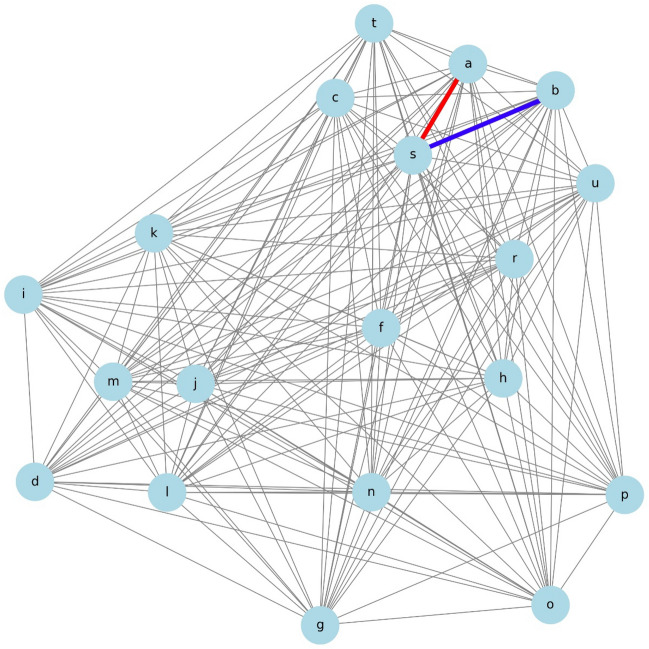
The shortest path from Δ1-hour pad test to TVT and VEL. A network diagram showing the paths connecting s: Δ1-hour pad test to a: TVT (in red) and b: VEL (in blue).

Figure 2 illustrates the shortest distances from a: TVT and b: VEL to s: Δ1-hour pad test. The paths depicted in the figure visually indicate that the distance from s to a (s-a) is shorter than from s to b (s-b), suggesting a: TVT as the recommended treatment.

According to the data, the correlation coefficient for s-a was approximately 0.437, whereas for s-b, it was approximately 0.396. These results clarify that a: TVT should be recommended for improving s: Δ1-hour pad test outcomes.

### Thought Experiment 2

The decision was made to determine whether TVT or VEL should be recommended for patients with SUI and UUI. A thought experiment was conducted to assess whether TVT (a) or VEL (b) should be recommended when the objectives are s:Δ1-hour pad test and u: ΔOABSS.

The following condition was added to the code of the heuristic function:

mandatory_nodes = ['c', 'r', 'j', 'i'].

optional_nodes = ['a', 'b'].

In Thought Experiment 2, it was run with no mandatory_nodes and optional_nodes = ['a', 'b'] # a:TVT and b:VEL.

**Figure 3:**
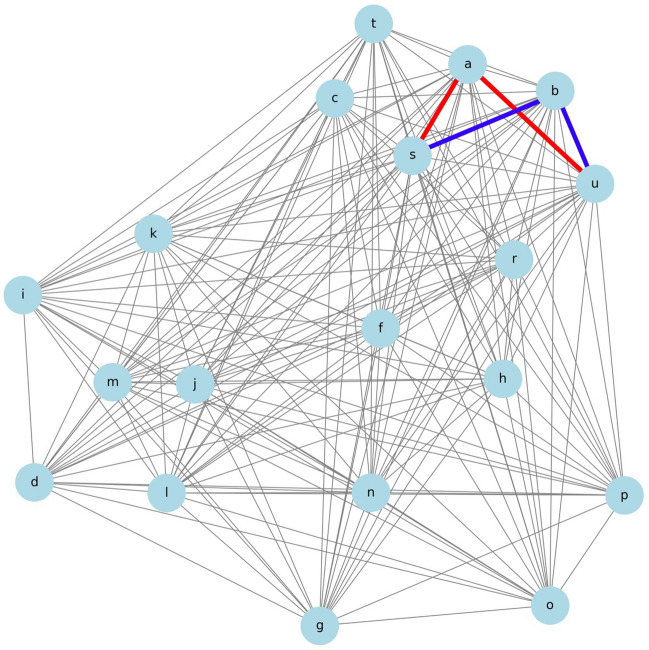
Shortest Path from Δ1-hour pad test to ΔOABSS. A network diagram depicting the shortest paths: one (in red) connecting s: Δ1-hour pad test to u: Δ OABSS via a: TVT, and another (in blue) linking a and s via b: VEL.

Figure 3 shows the shortest distances from a: TVT and b: VEL when targeting s:Δ1-hour pad test and u:ΔOABSS. The paths depicted in the figure led to the conclusion that a: TVT and b: VEL are equally recommendable, as the routes s-a-u and s-b-u appear to be of the same length visually.

The total correlation coefficient for s-a-u was approximately − 0.100, while for s-b-u, it was about 0.164. From these results, it is not accurate to say that both a: TVT and b: VEL should be equally recommended for s:Δ1-hour pad test and u:Δ OABSS. Instead, it became clear that b: VEL should be the recommended option. The positive correlation coefficient of b: VEL suggests a higher relevance to these objectives.

### Thought Experiment 3

This study explored which additional treatment, a: TVT or b: VEL, would be beneficial for individuals seeking to improve both s: Δ1-hour pad test and u: ΔOABSS outcomes, while necessarily undergoing c: PFMT. These individuals also had challenges related to r: desire for children, j: diabetes, and i: hypertension.

The following condition was added to the code of the heuristic function:

mandatory_nodes = ['c', 'r', 'j', 'i'] # c: PFMT, r: desire for children, j: diabetes, i: hypertension

optional_treatments = ['a', 'b'] # a: TVT and b: VEL.

**Figure 4:**
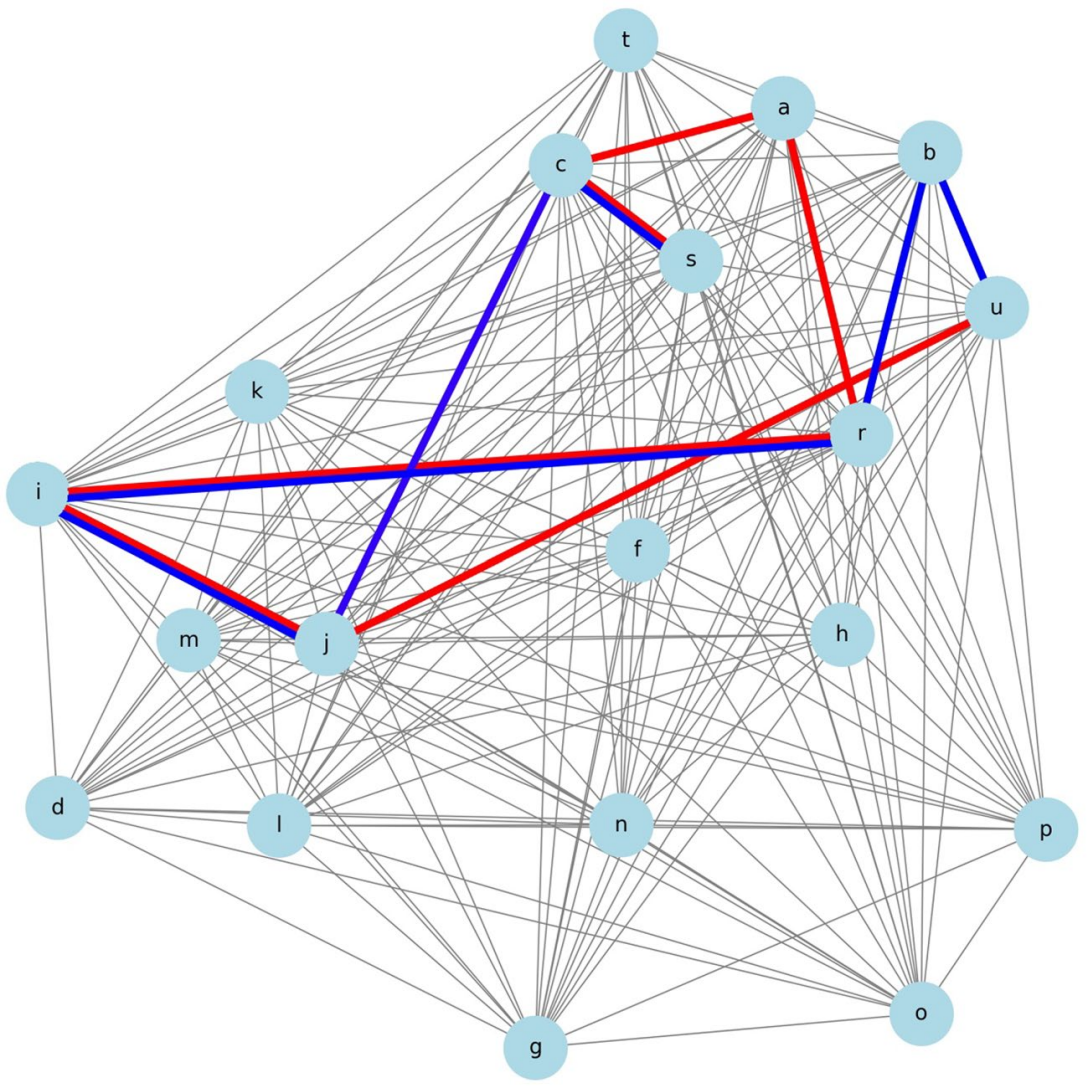
The shortest path encompassing various variables from Δ1-hour pad test to ΔOABSS. A network diagram illustrating the shortest paths. The first (in red) goes through a: TVT, including c: PFMT, i: hypertension, j: diabetes, and r: desire for children, connecting s: Δ1-hour pad test to u: ΔOABSS. The second (in blue) passes through b: VEL, also including c, i, j, and r, linking s: Δ1-hour pad test to u: Δ OABSS.

Figure 4 shows the shortest distance for this scenario.

Both paths were visually assessed to be of the same length, leading to the conclusion that a: TVT and b: VEL were equally recommended.

The paths including each treatment option and their respective lengths were as follows:

The shortest path including 'a: TVT' was in the following order:

Path: s: Δ1-hour pad test → c: PFMT → a: TVT → r: desire for children → i: hypertension → j: diabetes → u: ΔOABSS. The total length of this path was 3.973495479.

Conversely, the shortest path including 'b: VEL' was in this order:

Path: s: Δ1-hour pad test → c: PFMT → j: diabetes → i: hypertension → r: desire for children → b: VEL → u: ΔOABSS. The total length of this path was 3.935202947.

Based on these results, the path including "b: VEL" was slightly shorter, suggesting that VEL might be a more efficient treatment option for patients with specific characteristics and preferences described in Thought Experiment 3. However, it is important to note that this finding is based on a hypothetical scenario and may not be generalizable to all SUI patients. The actual treatment recommendation should be made on a case-by-case basis, considering each patient's unique clinical characteristics, preferences, and available evidence on the long-term effectiveness and safety of TVT and VEL.

Further research, including randomized controlled trials comparing TVT and VEL in various patient populations, is needed to validate these findings and provide more robust evidence to guide clinical decision making. Additionally, the development and validation of decision support tools incorporating graph theory and other mathematical approaches should be explored to enhance personalized treatment recommendations for SUI patients.

## Discussion

In this study, we adapted graph theory from discrete mathematics to explore the complex interactions between various variables related to SUI and treatments (TVT, VEL, PFMT). To our knowledge, this is the first time discrete mathematics has been applied to clinical medical treatment choices^5^.

First, the advantages of creating network diagrams using discrete mathematics were considered.

The generated network diagrams demonstrated the intricate interplay between health and personal variables. The dense clusters formed by lifestyle-related variables, such as smoking status, obesity, and hypertension, suggest strong interdependency among these factors. Valenzuela et al. explored a similar perspective in their research on obesity, cardiovascular risk, and lifestyle^27^. Additionally, the distinct subnetworks formed by reproductive health-related variables, such as the number of childbirths, menopausal status, and infertility treatments, indicate potential areas for future investigation. Scime et al. studied the overall risk of surgical menopause in women with a history of infertility treatment, focusing on reproductive health-related variables such as the number of childbirths, menopausal status, and infertility treatment^28^.

In this study, the research provided materials suggesting that for patients solely seeking improvement in the 1-h pad test, VEL is preferable for those aiming to improve both the 1-h pad test and OABSS. Furthermore, it appears to provide materials to judge whether VEL is recommended for individuals with multiple diseases or conditions who desire improvement in both the 1-h pad test and OABSS.

Second, the discussion centers on whether network diagrams can clarify the position of VEL treatment. It is important to note that the examples provided involve the introduction of a discrete mathematical approach, and for the reliability of the conclusions to be ensured, additional trials and sufficient randomized controlled trials on VEL are necessary.

Previous studies have demonstrated the effectiveness of VEL by Erel et al.^15^, Cervigni et al.^29^, and Fistonić et al.^30^. Considering that patients with SUI are often reported to have UUI, these recommendations for using VEL are considered realistic for SUI treatment^19,30^.

In this study, we simulated cases of individuals seeking to improve both "s: Δ1-hour pad test" and "u: ΔOABSS", who also have multiple issues such as "r: desire for children", "j: diabetes", and "i: hypertension". This reflects common scenarios encountered in clinical practice. Based on previous research, we were able to demonstrate which treatment, TVT or VEL, was superior for each variable. For instance, TVT showed superior results for certain variables, whereas VEL demonstrated good performance for different variables. When patients consider multiple variables related to their health condition and SUI, the navigation tool derived from this study aids in selecting the most suitable treatment method, taking these variables into account. Consequently, this enables data-based decision making to determine whether TVT or VEL is the optimal treatment for urinary incontinence.

Third, the heuristic approach of the graph theory utilized in this study was examined.

Heuristic methods have been applied in various contexts. Calculating the best driving route between multiple addresses involves considering distance, road conditions, traffic, and speed limits. Utilizing graph algorithms, such as smart heuristic functions and the A* algorithm, is key to effectively determining these routes^31^. Lai et al. used this method to calculate the walking paths of robots^26^. Oyelade et al. utilized it to identify alternative metabolic pathways for the malarial parasite Plasmodium falciparum, predicting potential drug targets^32^. Hosseini et al. presented a routing approach for ambulance patient transport, dealing with optimization problems considering the trade-off between wireless communication coverage and shortest path in ambulance routing choices^33^. Similarly, heuristic methods are employed in Google Navigation and for optimization of scheduling and air traffic control at airports^3,4,33^.

In this study, navigation utilized heuristic functions, a concept based on graph theory. Within this context, a heuristic approach is applied to determine the shortest path that includes the given conditions (mandatory nodes and optional treatments). The code generates multiple paths, including the specified nodes, and compares their lengths to select the shortest path. This process aims to find a reasonable solution efficiently rather than analyzing every possible path in detail.

Fourth, exploration focuses on the limitations of human visual perception in navigation using graph theory.

In this study, navigation using graph theory highlights the limitations of human visual perception. On the graph, the distances from "s: Δ1-hour pad test" and "u: ΔOABSS" to the treatments "a: TVT" and "b: VEL" appeared visually similar. However, the distance to VEL was shorter, making VEL the recommended treatment.

This phenomenon illustrates the gap between 'visual differences' and 'statistical significance.’ In clinical decision-making, while statistical significance is important, it is equally crucial that patients and healthcare providers intuitively grasp information. Integrating statistical analysis with intuitive understanding is considered the optimal approach for clinical decisions. The choice between treatments a and b should consider not only computational significance, but also side effects, costs, patient preferences, and lifestyle. This gap can be bridged using medical statistics and bioinformatics. Medical statistics provides statistical methods for quantitatively evaluating the effectiveness of treatments and analyzing the relationship between symptoms and treatments. Campo et al. offered practical guidance in teaching the magnitude of effect in physical therapy education, emphasizing the importance of distinguishing between statistical and clinical significance and interpreting research findings through effect size^34^. Bioinformatics combines biological data with statistical methods to analyze information related to disease diagnosis and treatment, making statistical findings more accessible and understandable through data visualization. Nammour et al. suggested that integrating data from information systems to enable clinicians to conduct their analyses without technical support is a multifaceted strategy for optimizing Best Practice Advisory alerts^35^.

Fifth, application examples anticipated in real-world clinical scenarios were examined. This item is divided into two issues: the problem of the environment for applying graph theory and the problem of individuals making decisions using graph theory. This section deals with environmental issues. In this field, the concern among many scholars is whether this theory can be adapted to both linear and nonlinear environments.

Graph theory can handle both linear and nonlinear relationships. Wei et al. proposed an approach to capture nonlinear relationships and interactions between nodes in complex networks, skillfully handling nonlinear features and complex data interactions^36^. Šutić et al. described the first solution to address the power flow problem using the Newton–Raphson method, which is a known approach for solving systems of nonlinear equations^37^. Zhao et al. used a graph convolution-based method to deal with complex periodic dependencies, nonlinearities, and spatial dependencies within the complex network of urban roads^38^. These examples demonstrate how graph theory can be applied to the analysis of complex networks with nonlinear relationships.

Several studies have reported nonlinearity in clinical medicine^39,40^. As indicated by the above evidence, even nonlinear databases in clinical medicine can be adapted to graph theory using several methods: (1) adjust the weights of edges using indicators other than correlation coefficients (e.g., mutual information) to reflect nonlinear relationships^41^. This allows for a more accurate representation of the strength of the nonlinear interactions between variables. (2) Combine graph theory with machine learning techniques, particularly graph neural networks (GNNs)^42,43^. GNNs can learn the complex nonlinear relationships between nodes and leverage this information for network analysis. (3) Dynamic network models were utilized to capture nonlinear relationships that change over time^44^. This enables the analysis of time-dependent interactions between variables, and provides a better understanding of disease progression and treatment effects.

Sixth, the discussion focuses on application examples in real-world clinical settings involving individual issues, namely, scenarios in which priorities emerge when proposing to patients.

In clinical scenarios, the diverse priorities of patients and physicians necessitate a nuanced approach to decision making^45,46^. For example, while some patients may prioritize resolving SUI, acknowledging potential risks, such as de novo urgency incontinence, the decision-making process must account for these varied concerns without oversimplification^47^. Graph theory offers a pathway for rapid yet sophisticated decision-making by incorporating (1) attribute-based and dynamic weighting of nodes, reflecting patient-specific attributes (e.g., number of childbirths, existing health conditions) and changing preferences through feedback^48^, and (2) multi-criteria decision analysis to evaluate a comprehensive spectrum of concerns (treatment efficacy, risks, quality of life)^49,50^. These strategies, tailored to individual patient needs and clinical data, aim to optimize treatment paths by considering both patient preferences and diverse health conditions. By dynamically adjusting the weights of connections between treatment options and integrating patient-reported outcomes, graph theory can significantly refine the clinical decision-making process, promoting patient-centered care and personalized treatment choices.

Finally, this study has several limitations.

1. The demographic focus on individuals aged 35–50 years may limit the generalizability of our findings across the broader SUI patient population, including older patients who may present with more complex clinical symptoms.
2. The exploration of treatment options was confined to TVT surgery and VEL, omitting a comprehensive analysis of other therapeutic modalities, such as pelvic floor muscle therapy, pharmacological treatments, and various surgical alternatives. This narrow focus might overlook the potential benefits or drawbacks of these unexamined treatments, potentially limiting the holistic understanding of SUI management.
3. The evidence supporting the efficacy and safety of TVT surgery is primarily derived from comparisons of different surgical techniques or materials rather than a direct comparison with minimally invasive treatments^51–53^. This may not fully reflect the contemporary ethical and clinical landscapes. Given modern ethical standards, this limitation becomes more complex when considering the difficulty of conducting randomized controlled trials (RCTs) that compare highly invasive procedures with less invasive alternatives given modern ethical standards.
4. Recent studies reporting promising results for VEL suggest that it is a viable and minimally invasive treatment option^14,54^. However, the long-term efficacy and safety of this treatment need to be established through further high-quality longitudinal studies. The comparison of the TVT and VEL, considering modern ethical concepts, seems to be feasible only through the analysis of large observational data.
5. The adoption of network graph theory in our study represents an innovative approach for integrating observational data for patient education. However, relying on this methodology introduces challenges related to potential oversimplification of complex clinical conditions and treatment dynamics. Our study's data-driven approach to weighting treatment options within the network graph highlights the need to consider the quality and representativeness of the data used as well as the definition of optimal treatment choices.
6. Future research must prioritize the validation of our graph-theory-based decision-making tool in clinical settings as a critical requirement. Transitioning from theoretical models to practical applications requires expanding the analyzed treatment options, incorporating diverse patient demographics, and extending follow-up periods to capture long-term outcomes. Integrating dynamic machine learning techniques and emphasizing patient-reported outcomes is crucial for enhancing the utility and relevance of the tool.

## Conclusion

In this study, we examined the application of graph theory to clinical decision-making for SUI treatment, focusing on 327 women aged 35–50. Utilizing data on TVT surgery and VEL treatment, we created network diagrams that detailed patient demographics, treatment methods, and outcomes, and elucidated the relationships between various health and demographic variables. We also showcase patient-specific TVT and VEL treatment choices on these diagrams, highlighting the shortest paths. Our findings underscore the importance of a data-driven, personalized approach to clinical decisions and demonstrate the potential of discrete mathematics in bridging the gap between mathematics and medicine, emphasizing its importance in enhancing clinical decisions and patient outcomes in SUI treatment.

## Data Availability

The data for this study can be downloaded from the following source: Okui, Nobuo, 2024, "A comparative analysis of urethral sling surgery and non-ablative vaginal Erbium:YAG laser treatment in 327 patients with stress urinary incontinence.", https://doi.org/10.7910/DVN/5OF6BV, Harvard Dataverse, V1.
